# Miscibility and ternary diagram of aqueous polyvinyl alcohols with different degrees of saponification

**DOI:** 10.1038/s41598-023-35575-w

**Published:** 2023-05-31

**Authors:** Junhyuk Kim, Shohei Ishikawa, Mitsuru Naito, Xiang Li, Ung-il Chung, Takamasa Sakai

**Affiliations:** 1 Department of Bioengineering, Graduate School of Engineering, The University of Tokyo, 7-3-1 Hongo, Bunkyo-Ku, Tokyo, 113-8656 Japan; 2 Department of Chemistry and Biotechnology, School of Engineering, The University of Tokyo, Tokyo, 113-8656 Japan; 3 Department of Materials Engineering, Graduate School of Engineering, The University of Tokyo, 7-3-1, Hongo, Bunkyo-Ku, Tokyo, 113-8656 Japan; 4 Faculty of Advanced Life Science, Hokkaido University, Sapporo, 001-0021 Japan

**Keywords:** Polymer chemistry, Materials science

## Abstract

Liquid–liquid phase separation (LLPS), an important phenomenon in the field of polymer science and material design, plays an essential role in cells and living bodies. Poly(vinyl alcohol) (PVA) is a popular semicrystalline polymer utilized in the synthesis of artificial biomaterials. The aqueous solutions of its derivatives with tuned degrees of saponification (DS) exhibit LLPS. However, the miscibility and LLPS behavior of PVA aqueous solution are still unclear. This study describes the miscibility diagram of the ternary mixture, where water and two types of poly(vinyl alcohol) (PVA) with different DSs [98 (PVA98), 88 (PVA88), 82 (PVA82), and 74 mol% (PVA74)] were blended. UV–Vis measurement was conducted to evaluate the miscibility. Immiscibility was more pronounced at elevated temperatures, exhibiting LLPS. The ternary immiscibility diagram, displaying miscible–immiscible behaviors in the aqueous mixtures of PVA74:PVA98, PVA82:PVA98, and PVA88:PVA98 (blended at a constant volume ratio), indicated that increasing the concentration, temperature, and blend ratio of PVAs at a lower DS increased immiscibility, suggesting that the free energy of mixing increases with increasing these parameters. The miscible–immiscible behaviors of PVAs/water systems provide fundamental knowledge about LLPS and the design of PVA-based materials.

## Introduction

Liquid–liquid phase separation (LLPS) is a thermodynamic physical phenomenon in which homogeneous solutions separate into distinct liquid phases with clear boundaries^1–3^. This phenomenon can occur in various solutions, including binary mixtures of organic solvents, polymer–solvent, polymer–polymer mixtures, and ternary polymer–solvent-nonsolvent mixtures^4,5^. LLPS has garnered attention in the fields of biology and soft-matter physics, where complex interactions between polymers^6^, proteins^7^, peptides^8^, and polysaccharides^9^ result in immiscible aqueous solutions. The stability of phases is governed by the Gibbs free energy of mixing Δ*G*_mix_ = Δ*H*_mix_ − *T*Δ*S*_mix_, where Δ*H*_mix_, *T*, and Δ*S*_mix_ are the enthalpy of mixing, absolute temperature, and entropy of mixing, respectively^2^. Phase separation occurs if Δ*G*_mix_ is positive^10^, and immiscibility is controlled by polymer concentration, molecular weight, temperature, and volume fraction^11^. Notably, LLPS plays an essential role in controlling the crystallization behaviors of polymers, such as blends of block-copolymer^12^, organic molecules^13^, and vanillin^14^. Therefore, a method to LLPS is the research direction in various scientific fields.

Poly(vinyl alcohol) (PVA) is a water-soluble synthetic polymer typically prepared from poly(vinyl acetate) (PVAc) via hydrolysis (also known as saponification). Moreover, per scientific knowledge since the 1960s, the saponification of PVAc proceeds with acceleration^15^, which can be interpreted in terms of the neighbor effect^16^. The blocky structure of VA-VAc copolymers, which is similar to that of PVA, has been confirmed by NMR^17,18^, demonstrating the importance of unit distribution in fully characterizing copolymer properties and understanding their behavior in different applications. PVA, a popular semicrystalline polymer, and is applied to the synthesis of hydrogels using its crystal as crosslinks^19–23^. Saponified PVAs have not only hydrophilic and crystalline (hydroxyl group) but also hydrophobic (acetate groups) moieties, making them immiscible or miscible aqueous solutions depending on the degree of saponification (DS)^23,24^. Control over the process of DS is essential for obtaining a strongly crystalized structure, because the miscible state in an aqueous solution determines the molecular interaction between PVAs. Several methods were developed for synthesizing mechanically robust PVA-based materials^25,26^. However, the miscibility of PVAs with tuned DS in water is still unclear, particularly in blended aqueous solutions of PVAs with different DS.

In this study, we investigated the miscibility of PVAs dissolved in water by tuning the blend ratio of saponified PVAs between DS = 98, 74, 82, and 88 mol% (PVA74:PVA98, PVA82:PVA98, and PVA88:PVA98), and developed ternary diagrams in terms of the blend ratio, concentration (*C*_PVA_), and temperature (*T*). Miscibility was evaluated by turbidity measured using UV–Vis spectroscopy at temperatures between 30 and 80 °C. Confocal laser scanning microscopy (CLSM) observation revealed the formation of phase-separated structures in the turbid ternary solutions at a certain blend ratio and temperature. The inferences drawn from the developed ternary diagram may accelerate the development of material design using PVAs.

## Results and discussion

### Evaluation of immiscibility of blended PVA solutions

**Figure 1:**
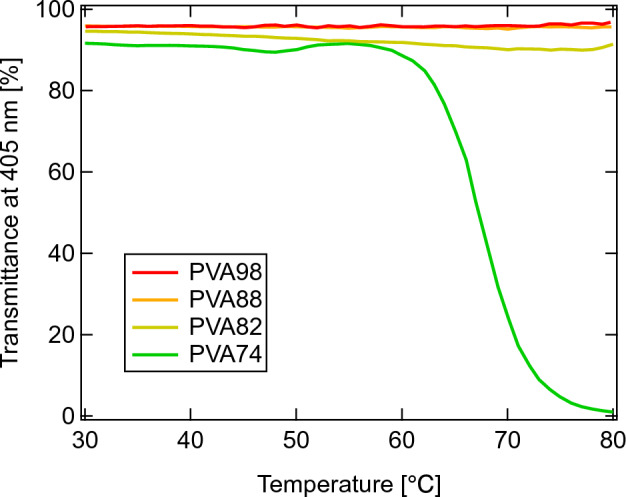
Temperature dependence of transmittance at 405 nm for PVA74 (green), PVA82 (brown), PVA88 (orange), and PVA98 (red). PVA solutions are prepared in water to obtain *C*_PVA_ = 10 wt%.

As the saponified PVAs contained both hydrophilic hydroxyl groups and hydrophobic acetate groups, their aqueous solutions exhibited amphiphilic behavior (Supplementary Fig. S1). The commercially available PVAs, including PVA98, PVA88, PVA82, and PVA74, possessed degrees of saponification that were mostly similar to those estimated in the ^1^H-NMR analysis. This finding is helpful as it facilitates discussion of the differences in DS (Supplementary Figs. S2, S3, S4, and S5). Prior to the evaluation of blended PVA solutions, we investigated the miscibility of pure PVA solutions using UV–Vis spectroscopy at controlled temperatures (Fig. 1).

Change in transmittance at *T* = 30–80 °C was negligible in pure PVA98 and PVA88 aqueous solutions, whereas that of PVA82 was marginal. However, the transmittance of PVA74 drastically decreased above *T* = 60 °C, exhibiting a lower critical solution temperature. This behavior is due to the increased fraction of the hydrophobic acetate group, which is consistent with the previous results^24^.

**Figure 2:**
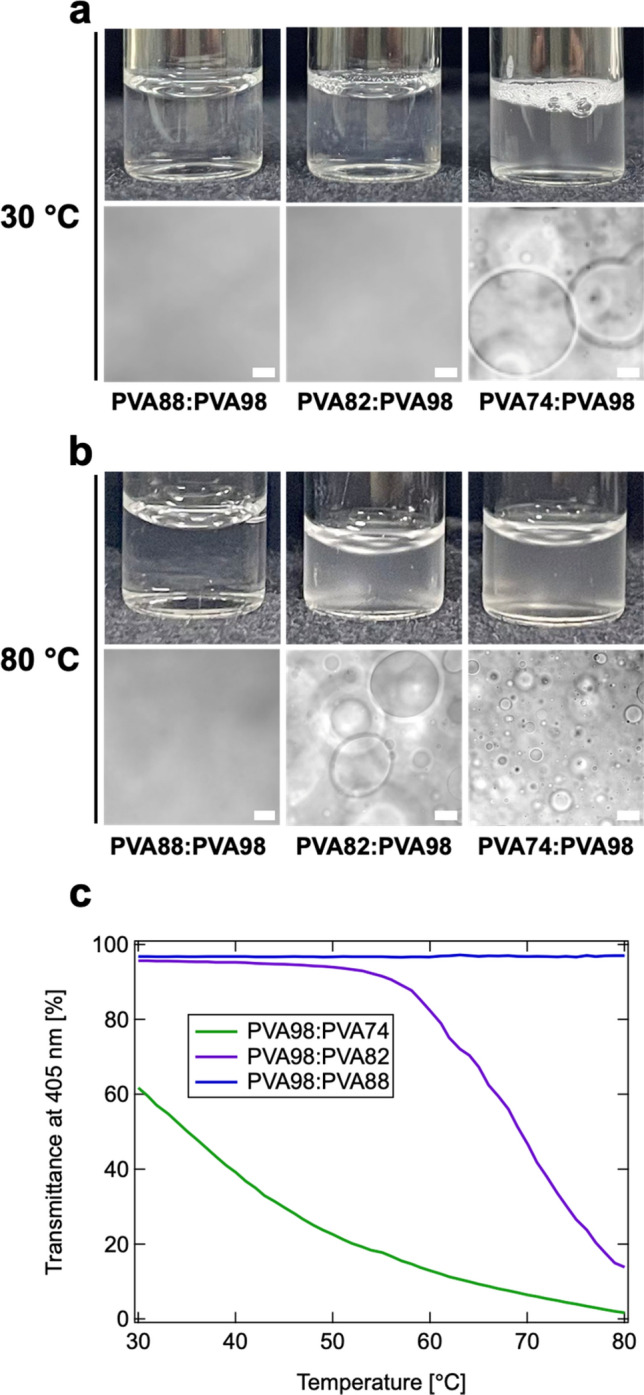
Phase-separation behavior of aqueous solutions of PVAs (weight ratio of 5:5) at *C*_PVA_ = 10 wt%. (**a**,**b**) Photographs (top) and confocal laser scanning microscopy (CLSM) (bottom) images of PVA88:PVA98 (left), PVA82:PVA98 (middle), and PVA74:PVA98 (right) incubated at *T* = 30 °C (**a**) and 80 °C (**b**). Scale bars indicate 20 µm. (**c**) Temperature-dependence of transmittance at 405 nm for PVA88:PVA98 (blue), PVA82:PVA98 (purple), and PVA74:PVA98 (green).

Next, we prepared PVA88:PVA98, PVA82:PVA98, and PVA74:PVA98 aqueous solutions at *C*_PVA_ = 10 wt% with a weight ratio of 5:5, and evaluated the immiscibility by visual inspection and CLSM at *T* = 30 and 80 °C (Fig. 2a,b). At *T* = 30 °C, PVA88:PVA98 and PVA82:PVA98 completely dissolved and formed transparent aqueous solutions. In contrast, PVA74:PVA98 was opaque and formed a precipitate, although the aqueous solution of pure PVA74 was transparent and miscible at that temperature and concentration. This immiscible behavior was also evident in the CLSM observations in the differential interference contrast (DIC) mode. The CLSM image of PVA74:PVA98 revealed phase-separated structures, similar to those in the immiscible polymer blends and LLPS^9,27–29^, which can occur through either spinodal decomposition or the nucleation and growth of PVA98-poor droplets. The mixtures of PVA88:PVA98 or PVA82:PVA98 did not contain any such structures even by CLSM at 30 °C. At 80 °C, PVA82:PVA98 and PVA74:PVA98 were immiscible (Fig. 2b) and displayed a phase-separated structure. In addition, the phase-separated structure in PVA74:PVA98 was finely dispersed at *T* = 80 °C compared to that at *T* = 30 °C. Similar to other systems^30^, the aqueous solutions of blended PVAs should separate into PVA74- and PVA98-rich phases. These results indicate that the miscibility of PVAs decreases with an increase in the difference in DS, and the phase-separation tendency is pronounced at a higher temperature.

To further investigate the phase-separation behavior, the blended PVA solutions were subjected to UV–Vis measurements during the cooling process from *T* = 80 to 30 °C (Fig. 2c). The change in transmittance was negligible in PVA88:PVA98 during the cooling process. However, in PVA82:PVA98 and PVA74:PVA98, the transmittance continuously increased with decreasing temperature. The PVA82:PVA98 became almost transparent at approximately *T* = 50 °C. Notably, the transmittance gradually increased as the temperature decreased even in PVA74:PVA98, strongly suggesting that the miscibility of PVA74:PVA98 was recovered at lower temperatures.

**Figure 3:**
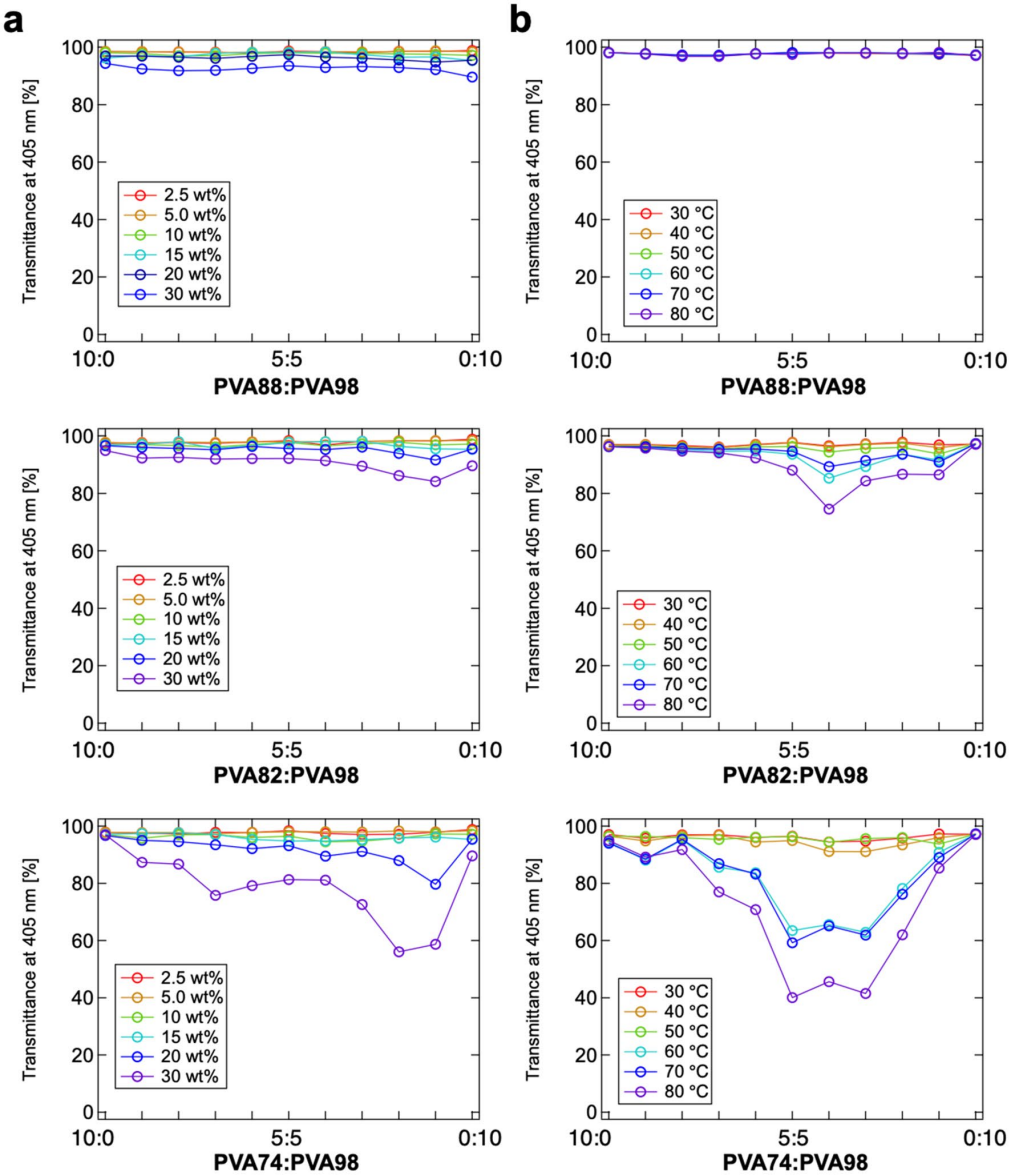
Transmittance at 405 nm of PVA88:PVA98 (top), PVA82:PV98 (middle), and PVA74:PVA98 (bottom) blended at weight ratios of 10:0, 9:1, 8:2, 7:3, 6:4, 5:5, 4:6, 3:7, 2:8, 1:9, and 0:10. (**a**) Concentration dependence from *C*_PVA_ = 2.5–30 wt% at *T* = 30 °C. (**b**) Temperature dependence from *T* = 30–80 °C with *C*_PVA_ = 10 wt%.

To further investigate the miscibility, the transmittance of the blended PVA solutions was evaluated in detail in terms of concentration (*C*_PVA_ was tuned in the range of 2.5–30 wt% at *T* = 30 °C) and temperature (*T* was tuned in the range of = 30–80 °C with *C*_PVA_ = 10 wt%) relative to the blend ratio (Fig. 3). At a constant temperature *T* = 30 °C, a higher *C*_PVA_ resulted in a lower transmittance even in PVA88:PVA98, where immiscibility was not observed by visual inspection or CLSM observation (Fig. 3a). Immiscibility in higher polymer concentrations is due to the elevated unfavorable interaction of PVAs with different DS owing to a significant positive Δ*H*_mix_^31^. Moreover, miscibility was low in the intermediate blend ratios for all temperatures, suggesting that Δ*H*_mix_ drives the phase separation. A similar trend was observed in the temperature dependence (Fig. 3b). The transmittance of PVA82:PVA98 and PVA74:PVA98 blended at approximately the weight ratio of 4:6 decreased with increasing temperature.

Hereinafter, the mixtures exhibiting a transmittance of below 90% acquired by UV–Vis measurements are defined as the immiscible state, which is commonly determined by the UV–Vis spectrum^27^ or visual inspection^32^. Thereafter, ternary diagrams, which show the phase behavior for the concentration, blend ratio, and temperature, are developed.

### Miscibility of blended PVA88:PVA98

**Figure 4:**
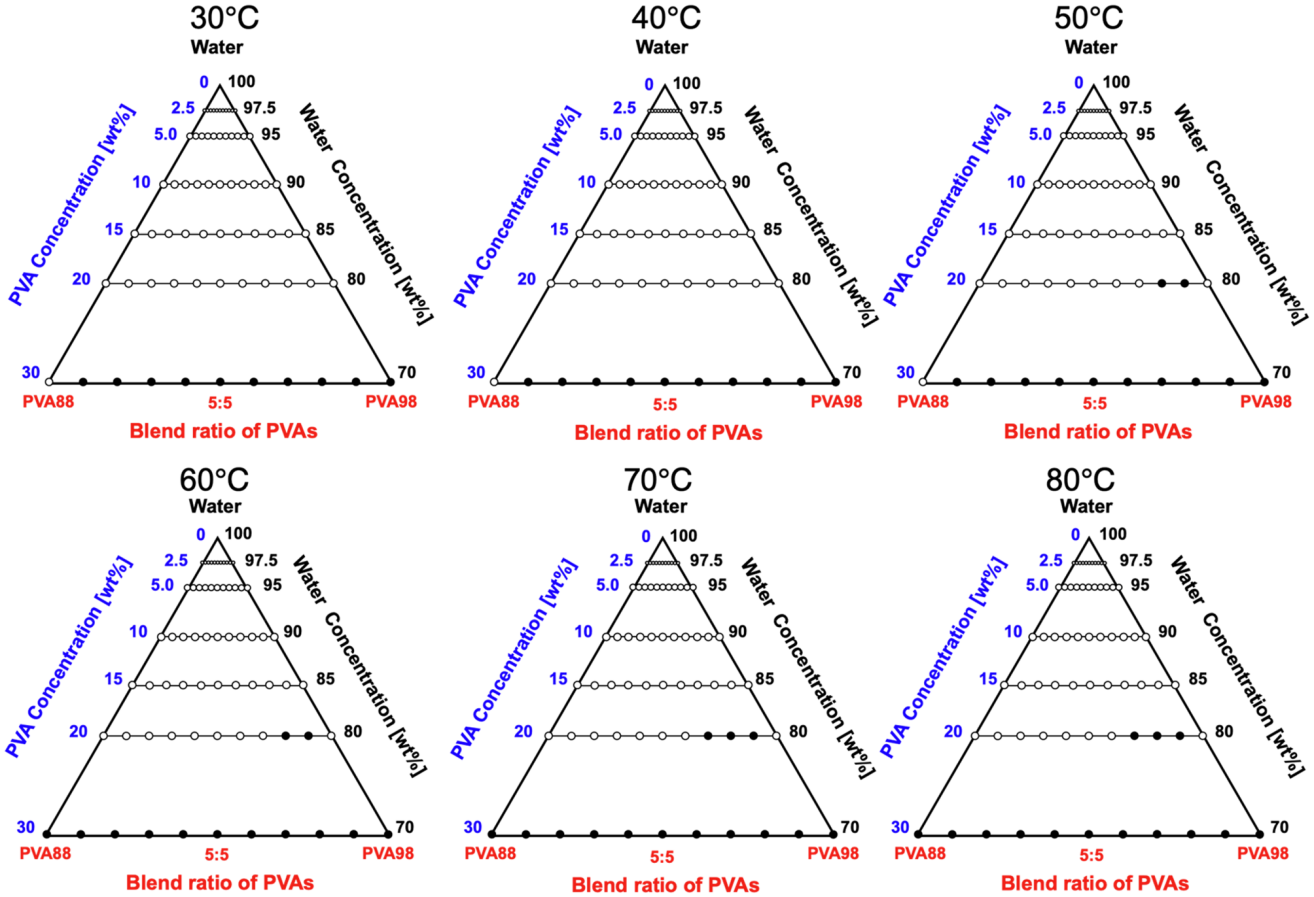
Ternary diagram of the PVA88:PVA98/water system incubated in the temperature range of *T* = 30–80 °C. The filled symbol represents the phase separation, while the open symbol represents the miscible state. The percentage value shows the concentration evaluated according to the weight ratio. The area enclosed in black is the region of immiscibility.

First, we developed a ternary diagram of PVA88:PVA98 (Fig. 4). The open and closed symbols indicate monophase and two-phase coexistence, respectively. We found that the system was entirely miscible at *C*_PVA_ ≤ 15 wt% regardless of the blending ratio. At *C*_PVA_ ≥ 20 wt%, phase separation was observed at specific blend ratios (2:8 and 1:9) under 50 °C, and the immiscible region subtly expanded to 3:7 at *T* ≥ 70 °C.

### Miscibility of blended PVA82:PVA98

**Figure 5:**
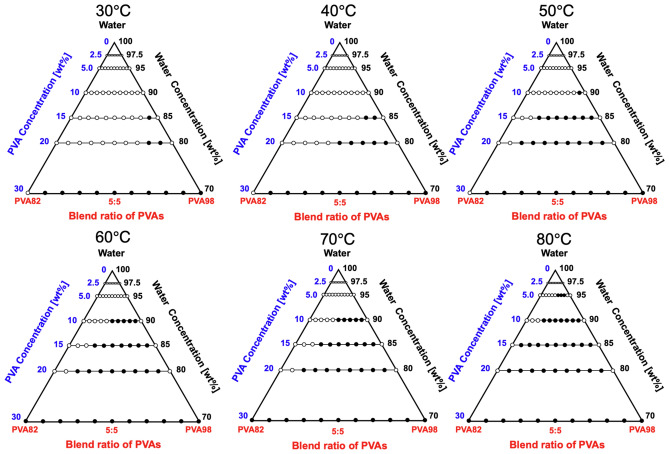
Ternary diagram of the PVA82:PVA98/water system incubated in the temperature range of *T* = 30–80 °C. The filled symbols represent immiscibility, while the open symbols represent miscibility. The percentage value shows the concentration evaluated according to the weight ratio. The area enclosed in black is the region of immiscibility.

The immiscibility of the PVA (PVA82:PVA98) aqueous solution was determined to be more pronounced because the difference in DS was increased from 10 (PVA88:PVA98) to 16 mol% (Fig. 5). The mixtures were completely miscible only at *C*_PVA_ = 2.5 wt% in the range tested. Further increase in *C*_PVA_ and/or temperature expanded the immiscible region, as with the case of PVA88:PVA98.

### Miscibility of blended PVA74:PVA98

**Figure 6:**
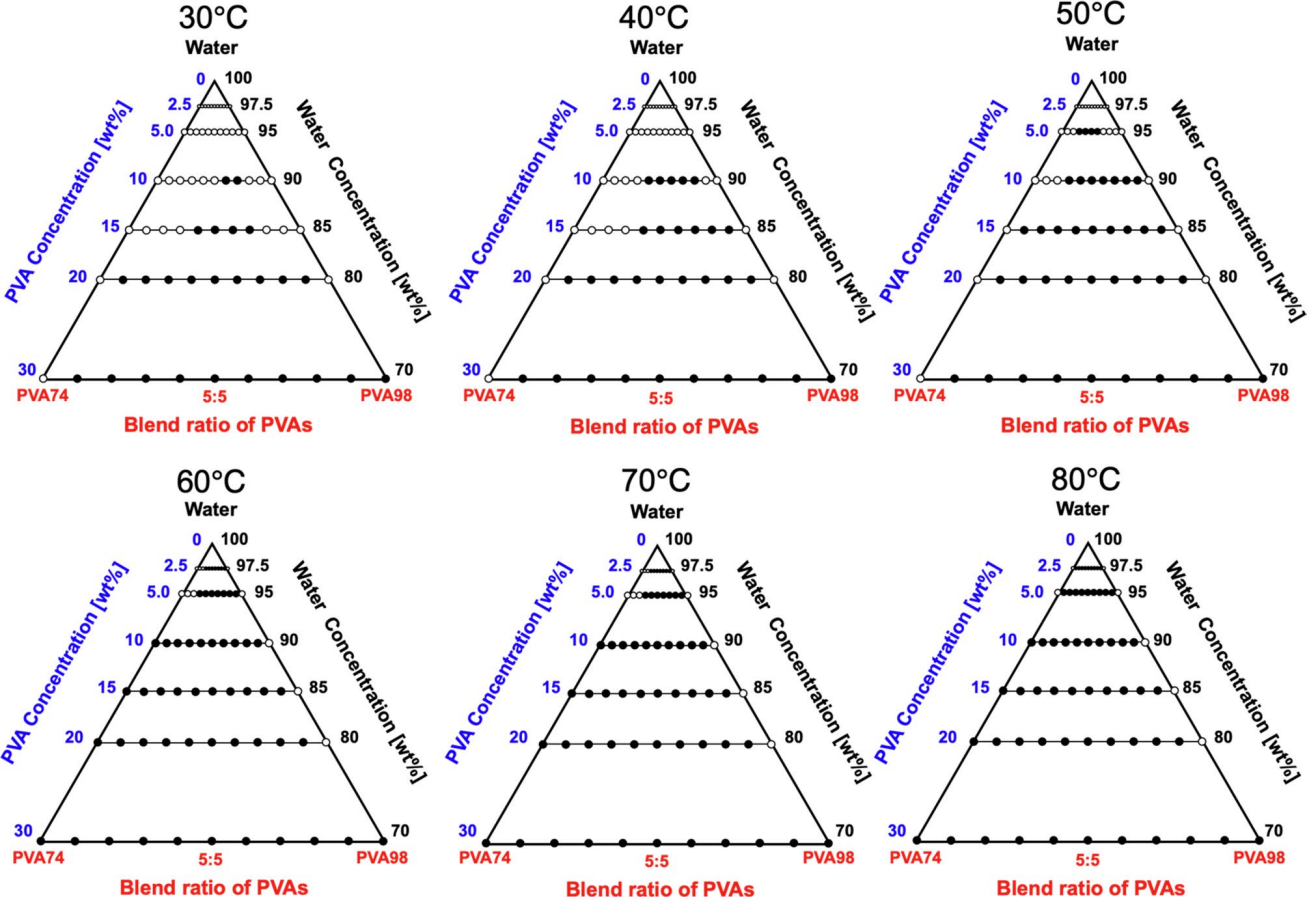
Ternary diagram of the PVA74:PVA98/water system incubated in the temperature range of *T* = 30–80 °C. The filled symbols represent immiscibility, while the open symbols represent miscibility. The percentage shows the concentration evaluated according to the weight ratio. The area enclosed in black is the region of immiscibility.

In the ternary diagram of the PVA74:PVA98 aqueous solution, the immiscible region was drastically broadened compared to other systems (Fig. 6). Even at *C*_PVA_ = 20 wt%, all blends regardless of the ratio exhibited miscibility at *T* = 30 °C. At *T* ≥ 50 °C, almost the entire region was immiscible, and no miscible region was observed at *T* = 80 °C. Notably, at 30 °C and 30 wt%, the binary mixtures of PVA88 and PVA74 with water were transparent, whereas the solution of PVA98 caused phase separation. This can be attributed to the excess hydroxyl groups on the PVA chains. At high concentrations, the hydroxyl groups promoted the formation of polymer aggregates through increased inter- or intra-chain hydrogen bonding. This aggregation leads to a state of insolubility and the emergence of a PVA-rich phase and a water-rich phase, culminating in a LLPS^33^.

### Immiscible diagram of PVA–PVA–Water ternary systems

**Figure 7:**
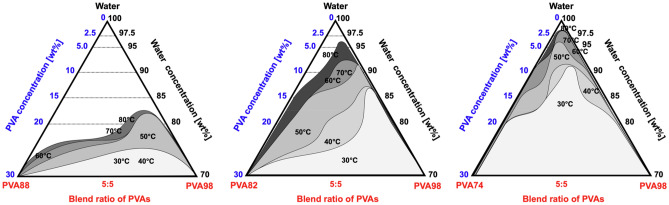
Inferred ternary diagram of blended PVA solutions incubated in the temperature range of *T* = 30–80 °C. The area enclosed in black is the region of immiscibility, and deeper colors represent higher temperatures.

Based on these experimental results, the immiscible diagram of PVA–water ternary systems were developed, as illustrated in Fig. 7. In the figure, the area enclosed by gray represents the immiscible zone. These diagrams include three essential points related to phase separation.

First, PVAs with different DS, even if the difference in DS is only 10 mol%, are recognized as different polymers and exhibit LLPS in the PVA–PVA–water ternary system. Notably, phase separation is also known to be triggered by mixing enantiomers^34^ and the polymers with different molecular weights^35^. Therefore, it is reasonable that even a tiny difference in DS (e.g., PVA98:PVA88) results in LLPS at specific ratios. Because the mixing of polymers is generally endothermic^2^, the difference in DS leads to a nonnegligible Δ*H*_mix_ and induces phase separation. Because such a minor difference in DS can cause phase separation, the relatively high polydispersity of PVAs (*M*_w_/*M*_n_ > 2.0, Supplementary Fig. S1) can induce the phase separation of binary PVA–water systems.

Second, the blended PVA solutions became immiscible at higher temperatures. As the entropy of mixing increases as the temperature increase, mixtures become miscible at higher temperatures. However, this is not the case, most probably because Δ*H*_mix_ increases with increasing temperature. The increased Δ*H*_mix_ at higher temperatures is indirectly supported by the lower critical solution temperature (LCST) behavior of pure PVA aqueous solutions^24^.

Third, phase separation is prone to occur at the blend ratio with a higher PVA98 concentration, with the phase boundaries approximately corresponding to pure PVA98. We could not determine the tie lines connecting the two phases in equilibrium in ternary systems^36^. This suggests that although PVAs are similar in composition, the affinity between PVA98 and water is stronger than the other interactions, potentially leading to a PVA98-rich phase and other PVA-rich phases.

The phase-separation behavior of the PVA–water binary system is complex, with factors such as blend ratio, concentration, and temperature playing crucial roles. Unlike traditional systems, which are often represented by two-branch binodal curves^37,38^, the curves in our phase diagrams exhibit multiple inflections. This complexity arises not from crystallization, but from the unique characteristics of our PVA systems, particularly the formation of polymer aggregates due to increased hydrogen bonding^33^. These aggregates lead to a state of insolubility, presenting as phase separation. The diagrams, based on extensive experimental data, capture this complexity and provide a more accurate representation of the observed results^17,39^, hence offering a comprehensive understanding of the phase behavior of the PVA–water system.

## Conclusion

In this study, we investigated the immiscible phase behavior of PVA–PVA–water ternary systems. The results demonstrated that even a subtle difference in the DS of PVAs, such as PVA98 and PVA88, caused phase separation, indicating that PVAs with different DS values are recognized as different polymers. We also found that the phase-separation affinity between the PVA- and water-rich phases was more substantial than the other interactions, resulting in a phase-separated structure reflected in LLPS with a distinct phase boundary. This study provides insights into the control of the miscible–immiscible behavior of PVA-based materials, their potential applications in hydrogel mechanical strength, and the control of the crystallinity of the PVA blends, which can be influenced by the miscible–immiscible behavior. Further thermodynamic analysis on Δ*H*_mix_ and Δ*S*_mix_ is required to develop a more comprehensive understanding of this ternary system. Overall, the findings contribute to the knowledge of LLPS and the design of PVA-based materials.

## Materials and methods

### Materials

Kuraray Poval™, the PVA (degree of polymerization = 550, DS = 74, 82, 88, and 98 mol%) for this study (Fig. S1), was procured from KURARAY Co. (Tokyo, Japan) and was used without further purification. Hereinafter, these PVAs with DS of 74, 82, 88, and 98 mol% were referred to as PVA74, PVA82, PVA88, and PVA98, respectively. D-PBS(−) (PBS) and D_2_O were purchased from FUJIFILM Wako Pure Chemical Corporation (Tokyo, Japan) and used without further purification.

### Preparation of PVA aqueous solutions

PVAs were dissolved in Milli-Q water at *T* = 94 °C and vigorously stirred for 2 h to obtain *C*_PVA_ = 2.5–30 wt%. The PVA solutions were then incubated at 25 °C for 2 h and blended at the same concentration in the following weight ratios: PVA74:PVA98, PVA82:PVA98, PVA88:PVA98 = 10:0, 9:1, 8:2, 7:3, 6:4, 5:5, 4:6, 3:7, 2:8, 1:9, and 0:10.

### Gel permeation chromatography

The PVAs were dissolved in PBS at 90 °C to obtain a *C*_PVA_ = 10 mg/mL. The PVA solutions were then filtered using a 0.45 μm filter (Sartorius AG, Göttingen, Germany). Size-exclusion chromatography was conducted with the JASCO HPLC system connected with two columns of Superose 6 increase and Superdex 75 increase (TOSOH Corporation, Tokyo, Japan). The flow rate was set constant at 0.6 mL/min, and the elution solvent was PBS with a 10 mM phosphate buffer and 150 mM NaCl.

### ^1^H-NMR

The PVAs were dissolved in D_2_O at 90 °C to obtain a *C*_PVA_ = 10 mg/mL. The ^1^H-NMR spectrum was evaluated by JNM-ECS400 (JEOL Ltd., Tokyo, Japan). The DS was calculated from the relative ratio of the integrated proton values between the CH_2_ protons and CH_3_ protons.

### Transmittance measurement of blended PVA solutions

Approximately 300 µL of the blended PVA solution was poured onto a 96-well plate and incubated in a water bath set at *T* in the range of 30–80 °C for 1 h. The transmittance of the blended PVA solutions in a 96-well plate was measured at 405 nm using a microplate reader (ARVO™ X3 microplate reader, PerkinElmer, Inc., Massachusetts, USA) to determine the miscibility–immiscibility of the blended PVA solutions.

Approximately 1000 µL of the pure and blended PVA solutions was poured into plastic cuvettes with an optical length of 10 mm. Temporal changes in the transmittance at 405 nm during the cooling process from *T* = 80 to 30 °C were measured using a spectrophotometer (V670 spectrophotometer, JASCO Corporation, Tokyo, Japan) every 0.5 °C/min.

### Microscopic observation of blended PVA solutions

The PVA solutions of PVA74:PVA98, PVA82:PVA98, and PVA88:PVA98 blended at a weight ratio of 5:5 were poured into a cylindrical silicone mold (diameter, 5 mm; height, 1 mm) and incubated at *T* = 30 or 80 °C for 1 h. The aggregate states of the PVAs were observed using a CLSM under the DIC mode (LSM 800, Carl Zeiss AG, Jena, Germany).

### Inferred miscibility diagram

Based on the transmittance results, a transmittance value less than 90% of that acquired by UV–Vis results was defined as the immiscible state, which is depicted as black circles. The plotted black circles were enclosed in a curvilinear line, and the inferred ternary miscibility diagrams were drawn using Keynote software (Apple, California, United states). Deeper black colors represent higher temperatures.

## Supplementary Information

Supplementary Information.

## Data Availability

The data that support the findings of this study available from the corresponding author on reasonable request.
