# Supplementary material for: Miscibility and ternary diagram of aqueous polyvinyl alcohols with different degrees of saponification
Source: Sci Rep. 2023 May 31;13:8791. doi: 10.1038/s41598-023-35575-w (PMC10232501; doi:10.1038/s41598-023-35575-w)
Supplement: Supplementary file 1 — Supplementary Information. [file 41598_2023_35575_MOESM1_ESM.docx]

**Supplementary information**

**Miscibility and Ternary Diagram of Aqueous Polyvinyl alcohols with Different Degrees of Saponification**

Junhyuk Kim^1,‡^, Shohei Ishikawa^2,‡,^*, Mitsuru Naito^3^, Xiang Li^4^, Ung-il Chung^1^, and Takamasa Sakai^2,^*

^1^Department of Bioengineering, Graduate School of Engineering, The University of Tokyo, 7-3-1 Hongo, Bunkyo-ku, Tokyo 113-8656, Japan

^2^Department of Chemistry & Biotechnology, School of Engineering, The University of Tokyo, Tokyo 113-8656, Japan

^3^Department of Materials Engineering, Graduate School of Engineering, The University of Tokyo, 7-3-1, Hongo, Bunkyo-ku, Tokyo 113-8656, Japan

^4^Faculty of Advanced Life Science, Hokkaido University, Sapporo 001–0021, Japan

‡These authors contributed equally to this work.

*Corresponding authors:

(S.I.) [ishikawa@gel.t.u-tokyo.ac.jp](mailto:ishikawa@gel.t.u-tokyo.ac.jp)

(T.S.) [sakai@gel.t.u-tokyo.ac.jp](mailto:sakai@gel.t.u-tokyo.ac.jp)

**

**Figure S1**. Chemical structure of PVAs. The degree of polymerization is 550, shown as n. The DSs are 74, 82, 88, and 98 mol%, implying that the ratios of x and y are 74:26, 82:18, 88:12, and 98:2, respectively.

**Figure S2**. ^1^H-NMR spectra of PVA98. **a** = *δ* 1.60 (m, 2H, CH_2_), **b** = *δ* 3.92 (m, 1H, CH), **c** = *δ* 2.00 (m, 3H, CH_3_).

**Figure S3**. ^1^H-NMR spectra of PVA88. **a** = *δ* 1.60 (m, 2H, CH_2_), **b** = *δ* 3.92 (m, 1H, CH), **c** = *δ* 2.00 (m, 3H, CH_3_).

**Figure S4**. ^1^H-NMR spectra of PVA82. **a** = *δ* 1.60 (m, 2H, CH_2_), **b** = *δ* 3.92 (m, 1H, CH), **c** = *δ* 2.00 (m, 3H, CH_3_).

**Figure S5**. ^1^H-NMR spectra of PVA74. **a** = *δ* 1.60 (m, 2H, CH_2_), **b** = *δ* 3.92 (m, 1H, CH), **c** = *δ* 2.00 (m, 3H, CH_3_).

**Figure S6**. GPC trace of PVA98 (black), PVA88 (red), PVA82 (blue), and PVA74 (orange). These traces were normalized so that the elution curves had the same peak intensity.
